# Retrovirus Entry by Endocytosis and Cathepsin Proteases

**DOI:** 10.1155/2012/640894

**Published:** 2012-12-06

**Authors:** Yoshinao Kubo, Hideki Hayashi, Toshifumi Matsuyama, Hironori Sato, Naoki Yamamoto

**Affiliations:** ^1^Department of AIDS Research, Institute of Tropical Medicine, Nagasaki University, Nagasaki 852-8523, Japan; ^2^Division of Cytokine Signaling, Graduate School of Biomedical Sciences, Nagasaki University, Nagasaki 852-8523, Japan; ^3^Pathogen Genomic Center, National Institute of Infectious Diseases, Tokyo 208-0011, Japan; ^4^Department of Microbiology, National University of Singapore, Singapore 117597

## Abstract

Retroviruses include infectious agents inducing severe diseases in humans and animals. In addition, retroviruses are widely used as tools to transfer genes of interest to target cells. Understanding the entry mechanism of retroviruses contributes to developments of novel therapeutic approaches against retrovirus-induced diseases and efficient exploitation of retroviral vectors. Entry of enveloped viruses into host cell cytoplasm is achieved by fusion between the viral envelope and host cell membranes at either the cell surface or intracellular vesicles. Many animal retroviruses enter host cells through endosomes and require endosome acidification. Ecotropic murine leukemia virus entry requires cathepsin proteases activated by the endosome acidification. CD4-dependent human immunodeficiency virus (HIV) infection is thought to occur via endosomes, but endosome acidification is not necessary for the entry whereas entry of CD4-independent HIVs, which are thought to be prototypes of CD4-dependent viruses, is low pH dependent. There are several controversial results on the retroviral entry pathways. Because endocytosis and endosome acidification are complicatedly controlled by cellular mechanisms, the retrovirus entry pathways may be different in different cell lines.

## 1. Introduction

 Retroviruses include many pathogenic agents in humans and animals. Human immunodeficiency virus (HIV) and human T-cell leukemia virus (HTLV) induce acquired immunodeficiency syndrome (AIDS) and adult T-cell leukemia (ATL), respectively. Murine leukemia viruses (MLVs) are also well-studied among retroviruses because the MLVs are used comparatively as animal models of several human diseases (leukemia, immunodeficiency, and neuropathogenic diseases) and as gene transfer tools. In addition, there are animal retroviruses that are important problems in the livestock industry, such as Visna, equine infectious anemia virus, bovine leukemia virus, and Jaagsiekte sheep retrovirus.

 Retroviruses contain envelope membranes consisting of lipid bilayers derived from virus-producing cells. Genomes of simple retroviruses such as MLVs encode three essential elements, gag, pol, and env genes. Complex retroviruses including HIV additionally encode accessory genes whose products regulate the retroviral expression and suppress host antivirus factors [1]. The gag and pol genes encode viral structural proteins and enzymes, respectively. These proteins are synthesized as precursor polyproteins and then are cleaved to mature peptides by a protease encoded by the retroviral pol gene. 

Retroviral envelope (Env) glycoprotein encoded by the env gene is also synthesized as a precursor protein and is cleaved to surface (SU) and transmembrane (TM) subunits by a cellular protease [2]. Retroviruses enter host cells by fusion between viral envelope and host cell membrane, following the recognition of cognate cell surface receptors. The SU protein binds to the cell surface receptor protein. The TM protein anchors the SU protein to the surface of viral particles and virus-producing cells by the complex formation of SU and TM. The TM protein mediates the membrane fusion reaction. The entry mechanisms of retroviruses are vigorously studied but are not completely understood. Elucidation of the retrovirus entry machinery would contribute to the development of new therapeutic approaches for retrovirus-induced diseases.

## 2. Membrane Fusion by Retroviral Env Glycoprotein 

**Figure 1:**
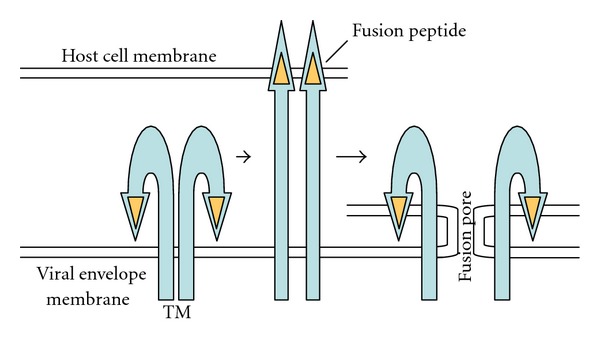
Conformational change of retroviral TM subunit for membrane fusion.

Mechanism of membrane fusion by the retroviral TM proteins is described elsewhere in details [3–7] and is similar to those used by envelope proteins of other enveloped viruses [8, 9]. Briefly, the retroviral entry mechanism is proposed as follows. The TM protein is thought to have hairpin-like structure (Figure 1). The binding of SU with its cognate cell surface receptor induces conformational changes of the TM subunit. The N-terminal hydrophobic domain of the TM subunit called fusion peptide is exposed by the conformational change and inserted into host cell membrane. The TM protein then coverts to a trimer-of-hairpins conformation, and viral envelope and host cell membranes approach and mix. Finally, the fusion pore is formed and expanded to derive the viral core into host cell cytoplasm. This conformational change pathway of the TM protein induces the membrane fusion for the retroviral entry into host cells.

## 3. Retrovirus Receptors

In this section, we will mainly focus on the infection receptors for MLV and HIV, with which entry mechanisms are most extensively studied among retroviruses. Other reviews should be referred to concerning the infection receptors of animal retroviruses in general [10, 11]. MLVs are divided into four groups according to their host ranges and infection interference, and the four groups recognize different cell surface receptors. Ecotropic MLVs infect mouse and rat and bind to cationic amino acid transporter 1 (CAT1) as the infection receptor [12]. Amphotropic MLVs infect many types of mammals, and inorganic phosphate symporter 2 (Pit2) is the amphotropic infection receptor [13, 14]. Polytropic MLVs has a similar host range to the amphotropic MLVs. The amphotropic MLVs cannot infect amphotropic virus-infected cells, because Pit2 are already occupied by the amphotropic Env proteins, called infection interference. Whereas the polytropic MLVs can infect amphotropic virus-infected cells, indicating that the polytropic virus receptor is different from the amphotropic receptor. Polytropic MLVs recognize XPR1 for the infection [15–17], whose physiological function is unknown yet. Xenotropic MLVs recognize the XPR1 as polytropic MLVs, but do not infect mouse cells. These MLV infection receptors are all multimembrane spanning proteins.

The infection receptors of HIV are CD4 and one of chemokine receptors (CXCR4 or CCR5) [18]. However, HIV variants that do not require CD4 for the infection are sometimes isolated from AIDS patients [19, 20] though the infectivity of CD4-independent variants is much lower than that of CD4-dependent viruses [21]. Such CD4-independent HIV variants recognize multimembrane spanning CXCR4 or CCR5 as the sole infection receptor, like the MLVs. CD4 is a single-membrane spanning protein, and HIV variants recognizing CD4 as the sole infection receptor have not been isolated. CD4-independent variants of simian immunodeficiency virus (SIV) are more frequently isolated than CD4-independent HIV variants [22, 23]. It is thought that CD4-independent HIV variants are prototypes of CD4-dependent HIVs [22–24].

## 4. C-Terminal Tail of Retroviral Env Protein Inhibits Membrane Fusion

 When retrovirus-producing and -susceptible cells are mixed, viral Env proteins on the cells can effectively interact with infection receptors on the neighboring susceptible cells via direct cell-to-cell contact. The interactions can have both positive and negative effects on the retrovirus replication. First, they can lead to cell-to-cell infection that allows very rapid and synchronized replication of virus compared to the cell-free infection [25, 26]. This can be advantageous for the virus replication in the presence of antiviral agents [27]. Second, the interactions can induce a negative effect, that is, the rapid apoptotic cell death, via syncytium formation [28–30]. This can be disadvantageous for the virus in that the sustained production of progeny virions becomes impossible. If the apoptotic cell death proceeded more efficiently than the virus replication, it eventually would result in poor progeny virus production. Therefore, it is conceivable that the retroviruses have some mechanisms to attenuate fusion capability of the envelope TM proteins in virus-producing cells and to primarily activate it in retroviral particles upon virion budding. Consistently, such mechanisms have been suggested for the Env TM proteins of MLV and HIV. 

In the case of MLV Env protein, C-terminal 16-amino acid peptide of the TM subunit called R peptide is further cleaved by the retroviral protease after the budding [31, 32]. The R peptide-containing Env protein is expressed in the virus-producing cells. The R peptide-truncated MLV Env protein can induce syncytia in susceptible cells, but the R peptide-containing Env protein cannot, indicating that the R peptide negatively regulates the syncytium formation of virus-producing cells [33, 34]. Viral particles carrying the R peptide-containing Env protein have much lower infectivity than those with the R peptide-cleaved Env, showing that the R peptide cleavage during virion maturation is required for the infectivity [35–37]. It has been reported that the R peptide controls the three-dimensional structure of the SU protein [38] and a disulfide bond between the SU and TM proteins [39], suggesting that the R peptide of TM subunit regulates the receptor-mediated SU conformational changes through the S–S bond between the SU and TM. It has been recently shown that the R peptide-cleaved TM forms separated Env legs, but the R peptide ties the TM legs together [40].

Although the C-terminal domain of the HIV TM protein is not cleaved, it is suggested that interaction between the HIV TM C-terminal region and Gag precursor protein suppresses the membrane fusion activity in virus-producing cells [41]. Processing of the HIV Gag precursor after budding abrogates the suppression of membrane fusion, and the mature virions gain sufficient fusion activity for the entry. The functions of C-terminal tails of retroviral Env proteins to inhibit membrane fusion are conserved among many retroviruses [42–45], though the mechanisms are different. The C-terminal domains of retroviral Env glycoproteins function to maintain the production of progeny virions by suppressing syncytium formation-directed apoptosis of virus-producing cells. 

## 5. PH-Dependent Retrovirus Infection

 Ammonium chloride, a weak base, neutralizes acid conditions in intracellular vesicles (Table 1). Concanamycin A and bafilomycin A-1 are specific inhibitors of the ATP-dependent proton pump/vacuolar ATPase (V-ATPase) that serves to acidify endocytic vesicles [46, 47]. To analyze the pH dependence of retrovirus entry, these compounds are frequently used. Additionally these inhibitors may affect trafficking of the intracellular vesicles, because siRNA-mediated knockdowns of subunits of V-ATPase complex affect trafficking of intracellular vesicles [48]. Previously it had been reported that ammonium chloride inhibits ecotropic MLV infection but does not amphotropic and xenotropic MLV infections, showing that ecotropic MLV infection occurs through acidic vesicles, but amphotropic and xenotropic MLV infections do not [49, 50] (Table 2). The more specific inhibitors of endosome acidification (concanamycin A and bafilomycin A-1) suppress all of ecotropic, amphotropic, polytropic, and xenotropic MLV infections [51, 52]. At present, it is generally accepted that ecotropic MLV infection requires acidification, because all the studies consistently reported the suppression of ecotropic virus replication with the inhibitors of endosome acidification. In contrast, it has been shown that xenotropic MLV infections are not suppressed by bafilomycin A-1 [53] (Table 2). Due to the controversial results, the entry pathway of xenotropic MLV is not clear yet. Because different cell lines were used in those reports, the low pH requirement of the xenotropic MLV infection may be dependent on the used cell lines (see below). 

 In case of avian leukosis virus (ALV) infection, there are also several controversial reports. The earlier reports show that ammonium chloride and bafilomycin do not affect ALV infection, suggesting that ALV infection does not require the acidification [54, 55]. In contrast, it has been recently reported that lowering the pH results in quick and extensive cell-cell fusion by ALV [56] and that the acidification inhibitors suppress ALV infection [57, 58]. It is now thought that receptor binding of ALV induces the Env protein to convert to its prehairpin intermediate at neutral pH [59, 60], and then endosome acidification triggers the formation of the final fusion-active form of the Env protein [61–63]. It has been proposed that the discrepancy came from unusual stability of the Env prehairpin intermediate, consequent ability of fusion to proceed upon washout of the acidification inhibitors after several hours, and the relatively high pH requirement for the outer leaflet mixing [64]. Finally, it is considered that ALV entry requires endosome acidification.

The acidification inhibitors suppress infections by mouse mammary tumor virus (MMTV) [65], foamy virus [66], equine infectious anemia virus (EIAV) [67, 68], Jaagsiekte sheep retrovirus (JSRV) [69], and enzootic nasal tumor virus [70]. These results suggest that infections by many animal retroviruses are low pH dependent.

## 6. Internalization Pathways

The requirement of low pH for the retrovirus infections reveals that retrovirus particles are internalized into acidic intracellular compartments during virus replication. There are several different pathways for the internalization of molecules; (i) phagocytosis, (ii) macropinocytosis, (iii) clathrin- and dynamin-dependent endocytosis, (iv) caveolin- and dynamin-dependent endocytosis, (v) lipid raft- and dynamin-dependent endocytosis, (vi) clathrin-, caveolin-, and dynamin-independent endocytosis that requires lipid raft, and (vii) dynamin-, clathrin-, caveolin-, and lipid raft-independent endocytosis [48, 71]. Here we will briefly summarize the accepted mechanisms and roles of internalization, relevant to the present review [48, 72, 73]. 

### 6.1. Phagocytosis

Specialized cells such as macrophages, neutrophils, and monocytes clear debris and pathogens by phagocytosis. Signaling cascades induce the actin rearrangement and form membrane extensions that cover the target particles and engulf it. Phagosomes become acidic by fusion with lysosomes (pH 5.0-6.0). Debris internalized by phagocytosis is degraded in the acidic phagosomes (phagolysosomes).

### 6.2. Macropinocytosis

Stimulation by certain growth factors or other signals causes membrane protrusions that fuse with the plasma membrane to form large intracellular vesicles known as macropinosomes that encapsulate large volumes of the extracellular fluid. Macropinosomes can either fuse with lysosomes (pH 5.0-6.0) or recycle back to the cell surface. There is no consensus as to the final fate of macropinosomes. Trafficking of macropinosomes seems to depend on cell type and mode of macropinocytosis induction.

### 6.3. Clathrin-Mediated Endocytosis

After ligands bind to their receptors, the receptor proteins are internalized into intracellular vesicles called endosomes. The endosome formation requires dynamin GTPase, and the endosomes are coated by clathrin proteins. Many receptors are segregated from their ligands in early endosomes due to weakly acidic condition (pH 6.0). Early endosomes become more acidic by V-ATPase-mediated acidification (late endosomes/lysosomes) (pH 5.0-6.0), and separated ligands are degraded by endosome proteases. Certain receptors are transferred from early endosomes to recycling endosomes (pH 6.4) and are reused on the plasma membrane. Some proteins are also recycled from late endosomes/lysosomes through the trans-Golgi network. Lysosomes often form multivescular bodies.

### 6.4. Caveolin-Mediated Endocytosis

Glycosylphosphatidylinositol (GPI)-anchored proteins, simian virus 40 (SV40), and cholera toxin trigger the formation of caveolae coated by caveolin proteins. These ligands are internalized into intracellular vesicles (pH 7.0) dependently on dynamin GTPase. The vesicles can be sorted to endosomes and become acidic. 

### 6.5. Clathrin- and Caveolin-Independent Endocytosis

Cholera toxin and SV40 can also be internalized via raft microdomains into GPI-anchored protein-enriched endosomes. Mechanisms regulating this internalization pathway are unclear as of yet.

## 7. Internalization of Retroviral Particles into Intracellular Vesicles

A dominant negative mutant of caveolin [74], siRNA-mediated knockdown of dynamin, and a dynamin inhibitor (dynasore) (Table 1) [52] suppress the amphotropic MLV infection, suggesting that amphotropic MLV particles are internalized by the dynamin- and caveolin-dependent endocytosis for productive infection (the fourth pathway). Ecotropic MLV particles are internalized into intracellular vesicles, but the vesicles are not colocalized with clathrin [75]. Furthermore, the dynamin-dominant negative mutant does not inhibit ecotropic MLV infection in human HeLa cells expressing the ecotropic MLV receptor, suggesting that ecotropic MLV particles are internalized by clathrin- and dynamin-independent endocytosis [75]. In contrast, another report indicates that siRNA-mediated knockdown of dynamin and dynasore suppresses ecotropic MLV infection in mouse NIH3T3, rat XC, and human TE671 cells expressing the ecotropic receptor [52] (Table 3). As mentioned above, the internalization pathway of ecotropic MLV might be dependent on the cell lines used. ALV [76] and EIAV [77] infections occur through clathrin-dependent endocytosis. JSRV infection required dynamin-dependent endocytosis [69]. Taken together, these reports strongly support a notion that infections by many animal retroviruses occur through endosomes and require endosome acidification. 

All of intracellular vesicles do not necessarily become acidic. For example, macropinosomes can be recycled to plasma membrane before their acidification, and recycling endosomes are formed from early endosomes and are transferred to plasma membrane [48]. Because many retroviral infections require endosome acidification, if viral particles are internalized into recycling endosomes, infectivity would decrease. To prevent this, the interaction between retrovirus Env proteins and the infection receptors is speculated to induce a signal to trigger the acidification of virion-containing intracellular vesicles.

## 8. Cleavage of Retroviral Env Proteins by Cathepsins

 Many retrovirus infections require endosome acidification. Influenza virus infection also requires endosome acidification, and treatment of influenza virus particles with low pH buffer activates its membrane fusion, indicating that low pH treatment directly induces conformational change of the influenza virus hemagglutinin to the fusion-active form. In contrast, low pH treatment of MLV particles does not activate the membrane fusion. Why does ecotropic MLV entry require endosome acidification? 

There is another mystery of the endosome-mediated infection. Proteins internalized into acidic late endosomes/lysosomes are generally degraded by endosome proteases including cathepsins. The acidification inhibitors suppress the degradation in late endosomes/lysosomes [47]. If the retroviral particles are degraded in late endosomes/lysosomes, the acidification inhibitors would enhance retrovirus infection. However, the acidification inhibitors rather suppress the infection [52]. Therefore, it is suggested that the retroviral particles incorporated into late endosomes/lysosomes are not degraded. Why are the retroviral particles not degraded in acidic late endosomes/lysosomes?

The finding that endosomal cathepsin proteases are necessary for the ecotropic MLV infection [78, 79] like Ebola virus infection [80] has provided a clue to understanding the questions. Because cathepsin proteases are activated by acidification, the ecotropic MLV entry into host cytoplasm requires cathepsin activation by acidification. The weakly acidic condition (pH 6) in early endosomes cannot activate cathepsin proteinases [81], suggesting that ecotropic MLV infection occurs via late endosomes/lysosomes. The acidification inhibitors suppress MLV infections by attenuating cathepsin protease activation. The evidence that the acidification inhibitors do not suppress the ecotropic MLV infection in active cathepsin-containing medium further supports this conclusion [52]. Our current model for entry of ecotropic MLV is that cathepsin proteases digest MLV Env glycoproteins to generate fusion-active forms rather than to break them up completely, because treatment of ecotropic and amphotropic MLV particles with cathepsin B protease results in a few digested products of the Env proteins but not their disappearance [52, 79]. It is still unclear how the MLVs are not degraded in the late endosomes/lysosomes by other proteases.

**Figure 2:**
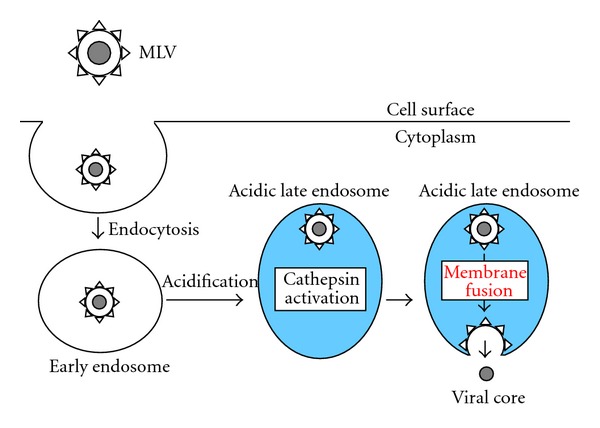
Entry pathway of ecotropic MLV in almost all susceptible cells. Blue area indicates acidic condition.

In summary, the entry pathway of ecotropic MLV occurs as follows (Figure 2). Ecotropic MLV particles are internalized into endosomes, following the interaction of Env protein with the infection receptor. The viral particle-containing endosomes become acidic by V-ATPase. Cathepsin proteases are activated in the acidic late endosomes. The activated cathepsins cleave the ecotropic Env proteins to confer them fusion active. The cleaved Env proteins induce fusion between the viral envelope and host cell endosome membranes. Finally, the ecotropic MLV cores enter into host cytoplasm.

 Although it is widely accepted that the ecotropic MLV infection requires endosome acidification and cathepsin proteases, the entry pathway of xenotropic MLV is not clear, because of the contradictory reports [52, 53]. We have shown that xenotropic MLV infection requires endosome acidification and cathepsin proteases like the ecotropic MLV infection [52]. In sharp contrast, the Liu research group has reported that inhibitors of endosome acidification and cathepsin proteases do not inhibit the xenotropic MLV infection [53]. Different cell lines used in these studies may induce different entry pathways of the xenotropic MLV. 

 Unlike the ecotropic MLV entry, it has been reported that a low-pH pulse of JSRV particles overcomes the bafilomycin-mediated infection inhibition [69], EIAV infectivity is enhanced by low-pH treatment [67], and cell-cell fusion induced by the ALV Env protein is enhanced at low pH [55]. Additionally, analysis of the pH dependence of the foamy virus Env-mediated fusion in a cell-cell fusion assay revealed an induction of syncytium formation by a short exposure to acidic pH [66]. The low-pH treatment of these retroviruses may directly induce the conformational changes of their Env glycoproteins to fusion active forms without the proteolytic cleavage, like influenza virus. 

## 9. PH-Independent MLV Infection in XC Cells

 Although the acidification inhibitors attenuate the ecotropic MLV infection in almost all susceptible cells [49, 52], the inhibitors have no effect on the ecotropic MLV infection specifically in rat XC cells, suggesting that the ecotropic MLV infection in XC cells is independent of low pH [49] (Table 2). Furthermore, the R peptide-containing ecotropic Env protein can induce pH-independent syncytium formation in XC cells, but cannot in other susceptible cells [82, 83]. By these results, it had been widely thought that ecotropic MLV entry into XC cells occurs at cell surface membranes and does not require the internalization of virions into intracellular vesicles and acidification. This XC cell-specific pH-independent ecotropic MLV infection was one of the well-known mysteries in the MLV field [84, 85]. We found that a cathepsin inhibitor, CA-074Me, efficiently suppresses the ecotropic MLV infection in XC cells, like in other susceptible cells, suggesting that the ecotropic MLV infection in XC cells requires endosomal cathepsin proteases [52]. This result is inconsistent with the previous theory that the ecotropic MLV infection in XC cells does not occur through endosomes. Because the ecotropic MLV infection requires cathepsin proteases activated by endosome acidification, the acidification inhibitors would be proposed to suppress the MLV infection by attenuating cathepsin activation. However, the acidification inhibitors do not reduce cathepsin activity in XC cells, but do so in other cell lines, suggesting that cathepsin proteases are activated without endosome acidification in XC cells [52]. XC cells do not express so much cathepsin that activation is sufficient at suboptimal pH, because cathepsin activity of XC cells is comparable to that of NIH3T3 cells. These results prompted us to speculate that the ecotropic MLV infection in XC cells occurs through endosomes. The result that dynasore and siRNA-mediated knockdown of dynamin expression suppress the ecotropic MLV infection in XC cells strongly supports this hypothesis.

**Figure 3:**
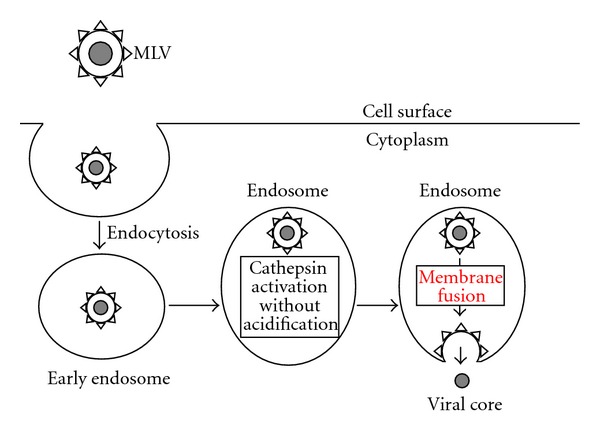
Entry pathway of ecotropic MLV in XC cells. Ecotropic MLV entry in XC cells may occur in acidic late endosomes, but endosome acidification is not required for the entry.

Taken together, the entry pathway of ecotropic MLV in XC cells is considered as follows (Figure 3). The ecotropic MLV particles are internalized into endosomes in XC cells, like in other susceptible cells. Cathepsin proteases are activated without endosome acidification. The activated cathepsins cleave the MLV Env protein, and the fusion between the viral envelope and host cell endosome membrane takes place for entry of the viral core into host cytoplasm. Because of the endosome acidification-independent activation of cathepsin proteases [52], the acidification inhibitors do not suppress the cathepsin protease activity and ecotropic MLV infection in XC cells. Additionally, this finding supports the above-mentioned hypothesis that the acidification inhibitors differentially affect retrovirus infections in different cell lines. The mechanism of acidification-independent cathepsin activation in XC cells is waiting to be resolved.

## 10. PH-Dependent Entry and PH-Independent Syncytium Formation by Retroviral Env Proteins

 The R peptide-cleaved MLV Env protein induces the fusion between the viral envelope and host cell membranes for viral entry and syncytium formation in susceptible cells [33, 34]. Cells expressing the R peptide-truncated Env protein behave as large MLV particles and fuse with neighboring susceptible cells. Therefore, the syncytium formation by the retroviral Env proteins is thought to represent the membrane fusion in retroviral entry. Because the syncytium formation by the retroviral Env protein may contribute to the development of degenerative disorders like AIDS [28, 29], and because an endogenous retroviral Env protein (syncytin) induces syncytiotrophoblast formation [86], the elucidation of mechanism of retroviral Env-induced syncytium formation is essential to understand retroviral pathogenesis and placenta development. The MLV entry into host cells is dependent on low pH, but the syncytium formation by the R peptide-truncated Env protein is independent [33]. Furthermore, the viral envelopes fuse with host cell membrane in endosomes [52, 75], but the syncytium formation appears to result from the fusion of cell surface membranes of the Env-expressing and host cells. In addition, the Env glycoprotein of a CD4-independent HIV efficiently induces pH-independent syncytium formation [87], but infection by CD4-independent HIV occurs through acidic endosomes [21] (see below). Multiple interactions between the viral Env and infection receptor proteins in much larger areas of cell-cell contact than virus-cell contact may abrogate the requirement of endocytosis for the membrane fusion. The finding that a cell adhesion molecule, LFA-1, facilitates HIV-mediated syncytium formation but not HIV infection supports this idea [88]. If the syncytium formation by the Env protein is independent of endocytosis, cathepsin proteases would be unnecessary for the syncytium formation. However, cathepsin inhibitors suppress syncytium formation by the ecotropic MLV Env protein [79]. Secreted cathepsin proteases may be involved in the pH-independent syncytium formation by the Env protein. Further study is needed to understand the mechanism of pH-independent syncytium formation by the retroviral Env proteins.

## 11. Endocytic Pathway of CD4-Dependent and -Independent HIV Entry

There are many controversial reports of the role of endocytosis in CD4-dependent HIV infection [94] (Tables 2 and 3). Early reports indicate that the acidification inhibitors enhance [89–91] or do not affect CD4-dependent HIV infection [92, 93], suggesting that the HIV does not enter into host cells via acidic vesicles. However, recent reports show that dynasore and chlorpromazine attenuate CD4-dependent HIV infection [95–97]. In addition, dominant negative mutants of dynamin and Eps15 inhibit CD4-dependent HIV infection [98]. Furthermore, analysis of localization of labeled HIV particles revealed that the HIV particles are internalized into intracellular vesicles [95, 99–102]. It has been reported that envelopes of HIV particles fuse with host cell membranes in intracellular vesicles by the following observation [95]. Envelopes of HIV particles were labeled with a hydrophobic fluorescent compound. When fusion of the labeled HIV envelope with host cell membrane occurs, the fluorescent compound is diluted and the fluorescent signals disappear. The vanishing of the fluorescent signals was observed in the intracellular vesicles but not at cell surfaces. These results suggest that HIV entry into the host cell cytoplasm may occur via endosomes. 

Interestingly, endosome acidification inhibitors attenuate infections by CD4-independent HIVs, which are thought to be prototypes of CD4-dependent viruses, suggesting that CD4-independent HIV entry may occur through acidic late endosomes, like many animal retroviruses [21]. The CD4-dependent HIVs can infect CD4-negative trophoblastic cells though the infection is 100 times less efficient than CD4-dependent Env-mediated infection [103]. HIV infection of trophoblasts forming the placental barrier may cause the mother-to-child transmission of HIV [104]. This infection occurs through an unusual entry pathway that is clathrin-, caveolin-, and dynamin-independent endocytosis requiring free cholesterol [71].

## 12. Degradation of HIV Particles by Endosome Proteases

Because acidification inhibitors enhance CD4-dependent HIV infection [89–91], HIV entry is independent of low pH, and the viral particles internalized into acidic late endosomes are degraded [105]. In other words, a proportion of HIV particles are internalized into acidic late endosomes although the internalization into late endosomes is not associated with the HIV productive infection. Consistently, the HIV particles appear to be internalized into acidic compartments shortly after inoculation into host cells [100]. 

**Figure 4:**
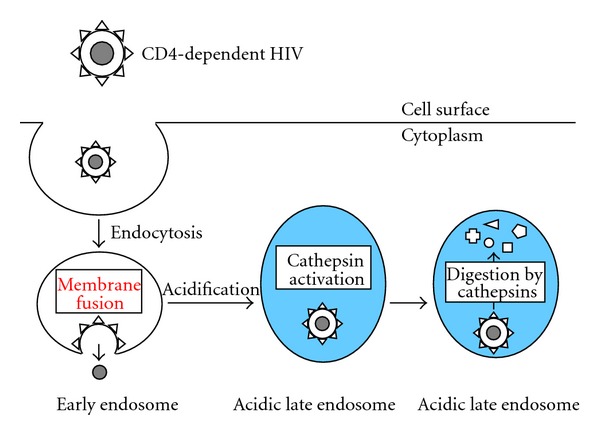
Entry pathway of CD4-dependent HIV. Blue area indicates acidic condition.

In summary, entry pathway of CD4-dependent HIV is considered as follows (Figure 4). The HIV particles are internalized into host cells by endocytosis, and the entry is independent of endosome acidification. HIV entry mainly occurs at early endosomes, and the HIV particles internalized into acidic late endosomes are degraded by endosome proteases.

**Figure 5:**
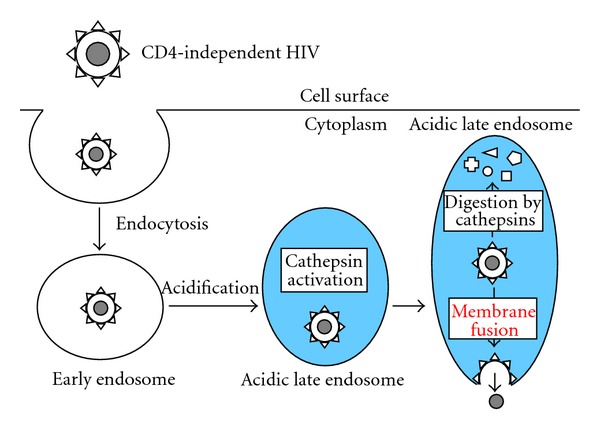
Entry pathway of CD4-independent HIV. Blue area indicates acidic condition.

It has been reported that a cathepsin inhibitor CA-074Me more significantly enhances CD4-independent HIV infection than CD4-dependent infection, and cathepsin protease activity in host cells is reverse-correlated with cellular susceptibility to the CD4-independent HIV infection [21]. These results suggest that CD4-independent HIV entry may occur at acidic late endosomes, and that viral entry competes with virion degradation by cathepsin proteases (Figure 5). 

Degradation by endosomal proteases in acidic vesicles following phagocytosis/macropinocytosis/endocytosis functions as an innate immune reaction against microbes to digest them and generate antigen peptides presented to helper T cells on MHC class II [106]. In fact, the activation of toll-like receptor signaling by LPS enhances cathepsin expression [21]. The CD4-dependent HIVs might evolve from CD4-independent viruses to overcome the endosome protease-mediated immunity. Some microbes express cystatin-like cathepsin inhibitors to protect themselves from the cathepsin-mediated immunity [107, 108]. Instead of having a cathepsin inhibitor, the CD4-dependent HIVs might gain the acidification-independent entry mechanism to protect from the endosome protease-mediated immunity.

In contrast to the CD4-dependent HIV entry pathway, ecotropic MLVs utilize these cellular innate immune reactions of endocytosis, acidification, and digestion by endosome proteases to enter into the host cell cytoplasm. By the ecotropic virus entry mechanism, the viruses can escape from these host immune reactions. It is suggested that the CD4-dependent HIV entry utilizes endocytosis, but not acidification and proteolysis by endosome proteases. The CD4-dependent HIV particles may be degraded by endosome proteases in acidic endosomes, and the infection titer is reduced [89, 91]. The CD4-dependent HIV Env proteins indeed contain several amino acid motifs that are digested by cathepsins [109, 110]. The ecotropic MLVs also have cathepsin-recognized amino acid motifs, but the digestion may activate the membrane fusion capability of the Env protein.

As mentioned above, the cathepsin inhibitor enhances CD4-independent HIV infection in cells with relatively higher level of cathepsin protease activity [21]. While, treatment of such cells with CA-074Me at higher concentration attenuates the CD4-independent infection. In addition, CA-074Me suppresses the CD4-independent HIV infection in cells with lower cathepsin activity (unpublished data). These results suggest that cathepsin proteases are required for the CD4-independent infection. Therefore, Env glycoproteins of the CD4-independent HIVs may be digested by cathepsin proteases to a fusion-active form, like the ecotropic MLV Env protein. Consistently, cathepsin proteases enhance CD4-dependent HIV infection and confer CD4-negative cells susceptible to CD4-dependent HIV infection [111–113]. Cathepsin-mediated digestion of CD4-dependent HIV Env protein may induce membrane fusion without CD4 binding. HIV particles in acidic endosomes are degraded by many endosome proteases including cathepsins. However, when the HIV Env proteins are digested only by a cathepsin, the infectivity may be enhanced.

## 13. Entry of Targeted Retroviral Vector

 Retroviral vectors are valuable tools in molecular biology research and human gene therapy. Several fundamental properties of retroviral vectors remain to be improved for effective gene transfer to specific target cells [114]. The effectiveness will be greatly enhanced, if their infection tropism is artificially modified to target specific cells [115]. There have been various attempts to establish redirecting infection tropism by genetically incorporating heterogenous ligands into the retroviral Env proteins [116–121]. However, retroviral vectors containing such modified Env proteins suffer from very low transduction efficiency or are not infectious. The redirected transductions of retroviral vectors with chimeric Env proteins are enhanced by the endosome acidification inhibitors, suggesting that the targeted vector particles internalized into acidic endosomes are degraded by endosome proteases [120, 122]. 

 Retroviral vectors carrying the ecotropic Env proteins chimeric with SDF-1*α* [123] and somatostatin [124] can transduce cells expressing CXCR4 and somatostatin receptor, respectively, as efficiently as retroviral vectors with the wild-type Env protein. It has not been examined whether efficient infections by the redirected retrovirus vectors occur through endosomes. Because the SDF-1*α*-chimeric Env protein appears to induce infection by the same mechanism as the wild-type Env protein [125], the redirected infection may occur through endosomes and require endosome acidification, like the wild type MLV Env protein. Elucidation of the entry pathways of these targeted retroviruses will likely contribute to the development of efficient cell lineage-specific retrovirus vectors.

## 14. Endocytic Entry of Ebola Virus-Pseudotyped Retrovirus Vector

 Retrovirus vectors can be pseudotyped with glycoproteins of various enveloped viruses. The pseudotyped retrovirus vectors enter into host cells by the entry mechanisms of the heterologous viral glycoproteins. Because the retrovirus vectors do not produce replication-competent viruses and the protocol is relatively simple, pseudotyped retrovirus vectors are widely used to identify entry pathways of various enveloped viruses [126–128].

**Figure 6:**
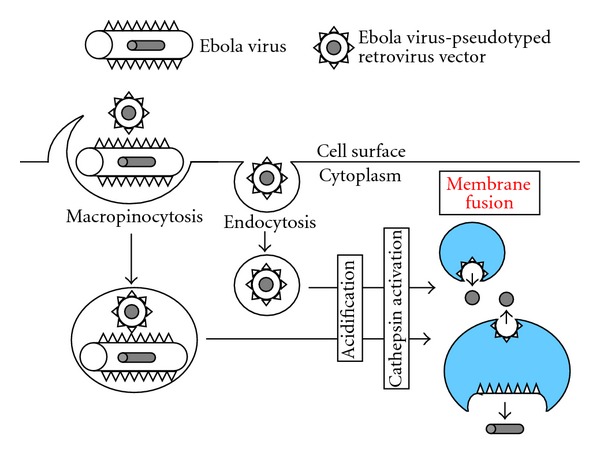
Entry pathways of Ebola virus and Ebola virus-pseudotyped retrovirus vector. Blue area indicates acidic condition.

 A dominant negative mutant of Eps15, siRNA-mediated knockdown of clathrin, and chlorpromazine suppress infection by an HIV vector pseudotyped with Ebola virus glycoprotein (GP), indicating that Ebola virus GP-mediated entry occurs through clathrin-dependent endocytosis [129]. Virion morphologies of the pseudotyped HIV vector and Ebola virus are much different. The pseudotyped HIV vector particles are round and the diameter is around 100 nm regardless of viral envelope glycoproteins. Whereas Ebola virus virions are long and filamentous as the name of filovirus should show. Typical clathrin-coated vesicles are large enough to incorporate the HIV vector particles, but not Ebola virus particles. Therefore, Ebola virus particles cannot be internalized into the endosomes. Does Ebola virus enter into host cells through endosomes? The finding that Ebola virus entry occurs via macropinosomes resolved this problem [130–133] (Figure 6). Macropinosomes have enough size to incorporate Ebola virus particles. However, entry of intact Ebola virus is still dependent on dynamin, which is not involved in classical macropinocytosis [133], and is partially inhibited by inhibitors of clathrin-dependent endocytosis [132]. In addition, it has been reported that the Ebola virus entry through macropinocytosis or endocytosis is dependent on the cell lines used [134]. Therefore, the entry route of Ebola virus is not clear yet. The Ebola virus infections via endocytosis and macropinocytosis both require acidification and cathepsin proteases [80, 135]. Although the pseudotyped retrovirus vector is useful to study the entry mechanism of viral envelope proteins, we should notice the possibility that entry pathway of the pseudotyped retrovirus vector is different from that of the original virus.

**Figure 7:**
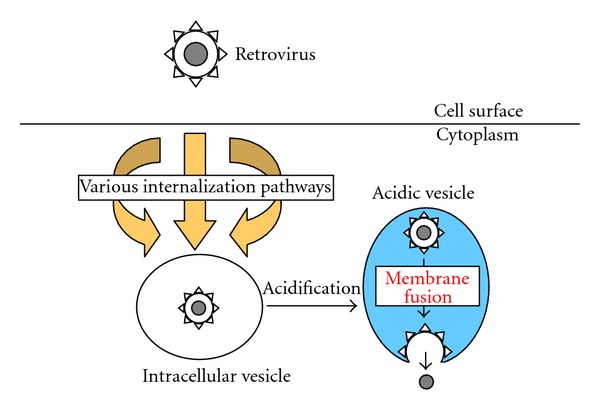
Retrovirus particles are internalized into intracellular vesicles by various pathways, and vesicle acidification is necessary for the infections.

 Size of macropinosomes is enough to incorporate not only Ebola virus particles but also pseudotyped HIV vector particles. Therefore, Ebola virus-pseudotyped HIV vector entry can occur through macropinocytosis (Figure 6). There is a report showing that HIV infection occurs through macropinosomes [102]. If host cells have both dynamin-independent macropinocytosis and -dependent endocytosis, the inhibition of dynamin function does not significantly affect the pseudotyped HIV vector infection. If host cells have endocytosis but not macropinocytosis, the inhibition of dynamin function severely suppresses the pseudotyped HIV vector infection. Retrovirus entry may be able to occur through several distinct internalization pathways for productive infection (Figure 7). This may be the reason why the inhibitors differentially affect retrovirus infections in different cells. Pathways of retrovirus internalization into intracellular vesicles may be unimportant for the productive infection. The GP of Ebola virus that enters host cells via macropinosomes can use endocytosis for the productive entry, when the retrovirus vector is pseudotyped with the Ebola virus GP. This result strongly supports the idea.

## 15. Conclusion

 Infections by many animal retroviruses occur through endosomes and require endosome acidification. The activation of cathepsin proteases by endosome acidification is required for ecotropic MLV infection. Whereas acidification directly induces conformational changes of several retroviral Env proteins to the fusion active forms. There are several internalization pathways of retrovirus particles, and the viral internalization pathways appear to be different in different cell lines. CD4-independent HIV infection may occur through endosomes and require endosome acidification, like other animal retroviruses. CD4-dependent HIV infection is thought to occur through endosomes but does not require endosome acidification. The CD4-dependent and -independent HIV particles are both degraded by endosome proteases, when the viral particles are internalized into acidic late endosomes. Retrovirus vectors pseudotyped with other viral envelope proteins are widely used to understand the entry mechanisms of the envelope proteins. However, entry pathway(s) of the pseudotyped retroviral vector could be different from that of the original virus.

 Retroviruses require cellular biological events of internalization, vesicle acidification, and cathepsin proteolysis for their entry into host cells. These biological events, especially in phagocytosis, function to protect host cells from microbe infection. Retroviruses utilize these immune reactions to enter into host cells. This entry mechanism of retroviruses is the best strategy to overcome the host immune attack, and many viruses other than retroviruses also enter into host cells by similar mechanisms [72, 136]. 

## Figures and Tables

**Table 1:** Inhibitors used in studies of retroviral entry pathway.

Inhibitors	Target
Ammonium chloride	Acidification of intracellular vesicles
Bafilomycin A-l	Acidification of intracellular vesicles
Concanamycin A	Acidification of intracellular vesicles
Dynasore	Dynamin-dependent endocytosis
Chlorpromazine	Clathrin-dependent endocytosis
CA-074Me	Cathepsin B protease
Dynamin DN mutant^1^	Dynamin-dependent endocytosis
Caveolin DN mutant	Caveolin-dependent endocytosis
Clathrin DN mutant	Clathrin-dependent endocytosis
Eps 15 DN mutant	Endocytosis

^1^DN: dominant negative.

**Table 2:** Differential dependence of HIV and MLV infections on endosome acidification.

Viruses	Dependence of acidification	Cell lines	Reference
Ecotropic MLV	Independent	Rat XC	[49, 52]
	Dependent	Mouse NIH3T3, human TE671	[49, 51, 52]

Amphotropic MLV	Independent	Mouse NIH3T3, rat XC	[49, 52]
	Dependent	Mouse NIH3T3, human TE671	[51, 52]

Polytropic MLV	Independent	Rat XC	[52]
	Dependent	Mouse NIH3T3, human RE671, rat XC	[52]

Xenotropic MLV	Independent	Human HT1080, HTX, porcine, rat XC	[49, 50, 52, 53]
	Dependent	Mouse NIH3T3, human RE671	[52]

CD4-dependent HIV	Independent	Human CEM, HeLa, C8166, VB	[49, 89–93]
	Independent	Human 293T, HeLa, TE671	[21]

CD4-independent HIV	Dependent	Human 293T, HeLa, TE671	[21]

**Table 3:** Differential internalization pathways of HIV and MLV infections.

Viruses	Internalization pathway	Cell lines	Reference
Ecotropic MLV	Dynamin dependent	Mouse NIH3T3, human TE671, rat XC	[52]
	Dynamin-, clathrin independent	Human HeLa	[75]

Amphotropic MLV	Dynamin dependent	Mouse NIH3T3, human TE671, rat XC	[52]
	Caveolin dependent	Mouse NIH3T3	[74]

Polytropic MLV	Dynamin dependent	Mouse NIH3T3, human TE671, rat XC	[52]

Xenotropic MLV	Dynamin dependent	Mouse NIH3T3, human TE671, rat XC	[52]

CD4-dependent HIV	Dynamin dependent	Human HeLa	[95]
	Clathrin dependent	Human primary T lymphocyte	[95–97]
	Dynamin-, Eps15 dependent	Human HeLa	[98]
	Dynamin-, Eps15 independent	Human 293T, HeLa, TE671	[21]

CD4-independent HIV	Dynamin-, Eps15 dependent	Human 293T, HeLa, TE671	[21]
